# Updated sesame genome assembly and fine mapping of plant height and seed coat color QTLs using a new high-density genetic map

**DOI:** 10.1186/s12864-015-2316-4

**Published:** 2016-01-05

**Authors:** Linhai Wang, Qiuju Xia, Yanxin Zhang, Xiaodong Zhu, Xiaofeng Zhu, Donghua Li, Xuemei Ni, Yuan Gao, Haitao Xiang, Xin Wei, Jingyin Yu, Zhiwu Quan, Xiurong Zhang

**Affiliations:** Oil Crops Research Institute of the Chinese Academy of Agricultural Sciences, Key Laboratory of Biology and Genetic Improvement of Oil Crops of the Ministry of Agriculture, Wuhan, 430062 China; Shenzhen Engineering Laboratory of Crop Molecular Design Breeding, BGI-agro, 518083 Shenzhen, China

## Abstract

**Background:**

Sesame is an important high-quality oil seed crop. The sesame genome was *de novo* sequenced and assembled in 2014 (version 1.0); however, the number of anchored pseudomolecules was higher than the chromosome number (2n = 2x = 26) due to the lack of a high-density genetic map with 13 linkage groups.

**Results:**

We resequenced a permanent population consisting of 430 recombinant inbred lines and constructed a genetic map to improve the sesame genome assembly. We successfully anchored 327 scaffolds onto 13 pseudomolecules. The new genome assembly (version 2.0) included 97.5 % of the scaffolds greater than 150 kb in size present in assembly version 1.0 and increased the total pseudomolecule length from 233.7 to 258.4 Mb with 94.3 % of the genome assembled and 97.2 % of the predicted gene models anchored. Based on the new genome assembly, a bin map including 1,522 bins spanning 1090.99 cM was generated and used to identified 41 quantitative trait loci (QTLs) for sesame plant height and 9 for seed coat color. The plant height-related QTLs explained 3–24 % the phenotypic variation (mean value, 8 %), and 29 of them were detected in at least two field trials. Two major loci (*qPH-8.2* and *qPH-3.3*) that contributed 23 and 18 % of the plant height were located in 350 and 928-kb spaces on Chr8 and Chr3, respectively. *qPH-3.3*, is predicted to be responsible for the semi-dwarf sesame plant phenotype and contains 102 candidate genes. This is the first report of a sesame semi-dwarf locus and provides an interesting opportunity for a plant architecture study of the sesame. For the sesame seed coat color, the QTLs of the color spaces L*, a*, and b* were detected with contribution rates of 3–46 %. *qSCb-4.1* contributed approximately 39 % of the b* value and was located on Chr4 in a 199.9-kb space. A list of 32 candidate genes for the locus, including a predicted black seed coat-related gene, was determined by screening the newly anchored genome.

**Conclusions:**

This study offers a high-density genetic map and an improved assembly of the sesame genome. The number of linkage groups and pseudomolecules in this assembly equals the number of sesame chromosomes for the first time. The map and updated genome assembly are expected to serve as a platform for future comparative genomics and genetic studies.

**Electronic supplementary material:**

The online version of this article (doi:10.1186/s12864-015-2316-4) contains supplementary material, which is available to authorized users.

## Background

The sesame (*Sesamum indicum*) is a member of the family Pedaliaceae and order Lamiales [1] and is a diploid species with 13 pairs of chromosomes (2n = 2x = 26). In our previous study, the sesame genome was estimated to be approximately 357 Mb in size based on *de novo* sequencing of Zhongzhi No. 13. The assembled genome (version 1.0) was predicted to contain 27,148 protein-coding genes. The version 1.0 assembly anchored 150 scaffolds into 16 pseudomolecules that harbored 85.3 % of the assembled genome and 91.7 % of the predicted genes [2, 3]. We attempted to anchor additional scaffolds and contigs to improve the sesame genome assembly and equalize the number of sesame chromosomes. To achieve this goal, a high-quality and dense genetic map is essential. Although two sesame genetic maps have been published [4, 5], they contain more than 13 linkage groups. The numbers of breeding lines and temporary populations usually contribute to the construction of genetic maps [6].

Genetic maps are useful for discovering, dissecting, and manipulating the genes responsible for simple and complex traits in crop plants [7]. A high-quality genetic map not only improves genome assembly but also provides a foundation for mapping the genes or quantitative trait loci (QTLs) that underlie agronomic traits of important oil crops such as the sesame. The sesame seed is useful because of its high oil content and quality. The sesame antioxidative furofuran lignans such as sesamin, are the focus of research in medicine and pharmacology due to their potent pharmacological properties including the ability to decrease blood lipid [8] and cholesterol [9] levels. Moreover, the diploid characteristic, high oil content, small genome size, and smaller number of lipid-related genes distinguish sesame from other polyploid oil crops such as soybean, peanut, and rapeseed and make it a valuable plant model to study oil biosynthesis and various traits [2]. Despite the importance and long history of cultivation of the sesame, few gene loci have been fine-mapped due to a lack of genetic and genomic studies and gene cloning and functional analyses.

Plant architecture is crucial for the grain yield and is determined by the plant height (PH) and other traits [10]. The “green revolution” was characterized by breeding semi-dwarf or dwarf wheat and rice cultivars to achieve an increased harvest index and improved adaption to the irrigated and fertile environments usually inhabited by taller plants [11–13]. Sesame has a low yield capacity compared to other crop plants due to its high PH, low harvest index, and susceptibility to biotic and abiotic stressors [14]. Mutant sesame lines with a determinate growth habit have been generated to reduce the sesame PH. Unfortunately, this characteristic was linked to short fruiting zone length and low yield [14, 15] phenotypes; thus, new dwarf or semi-dwarf sesame genetic resources must be explored.

Another important sesame trait is the seed coat color which varies widely from white to black. Black sesame seeds are favored as food and medication in Asia, whereas white sesame seeds are primarily used to produce oil. Some studies have shown that white sesame seeds typically have higher sesamin or sesamolin content [16], whereas black sesame seeds usually have higher ash and carbohydrate content and lower protein, oil, and moisture ratios [17].

Single nucleotide polymorphisms (SNPs) are the most abundant small-scale form of genetic variation in humans and plants and SNP markers have become increasingly important tools for molecular genetic analyses [18, 19]. Restriction-site associated DNA sequencing (RAD-seq) using next generation sequencing platforms reduces the representation of the genome and allows oversequencing of the nucleotides adjacent to restriction sites; thus, it is a relatively cost-effective and precise method for the detection of SNPs [20]. In this study, we constructed a recombinant inbred line (RIL) population consisting of 430 lines using a newly discovered semi-dwarf sesame strain. A new high-density sesame genetic map with 13 linkage groups was prepared using RAD-seq which enabled us to update the sesame genome assembly to a new level and to finely map important agronomic traits such as the PH and seed coat color.

## Results and discussion

### Construction of a new sesame genetic map

We RAD sequenced the 430 RILs and the two parental lines and generated more than 1.7 × 10^8^ paired-end reads. Each line produced a mean of 76.5 Mb of high-quality sequence data. All reads were aligned with the Zhongzi No. 13 scaffold sequence which has a total effective genome length of 274 Mb based on analysis using BWA software [21], and 90.97 % of the reads were mapped onto reference sequences across the various lines. Approximately 5.4 % of the reference genome was tagged by RAD-Seq, with a mean value of 4.53-fold coverage.

**Fig. 1:**
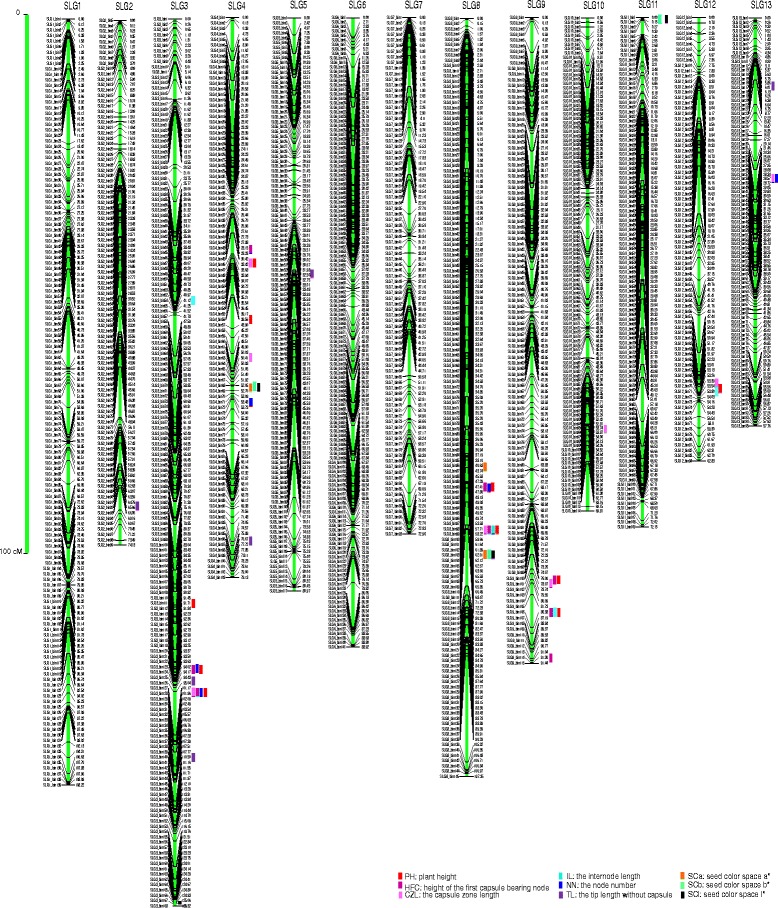
High density genetic map of the sesame genome and the mapped QTLs. Positions of the mapped QTLs for plant height (PH), capsule zone length (CZL), height of the first capsule-bearing node (HFC), internode length (IL), node number (NN), and tip length without the capsule (TL). The seed coat color space L*, a*, and b* values are indicated with colored rectangles centered at the peak of each QTL

The tags from the two parents Zhongzhi No. 13 and ZZM2748 were subjected to comparative analysis to detect SNPs. After filtering out poor SNPs and those with a significantly distorted segregation ratio (*P* < 0.01), 11,924 SNPs were retained for genotyping of the RIL population. Due to the limitation of markernumbers that can be analyzed in JoinMap software, a portion of the SNPs was selected to construct the primary genetic map. Based on the primary genetic map, we rearranged the scaffolds from the previous genome assembly and generated a new sesame genome sequence. Then, a bin map was constructed by joining the consecutive intervals on the genome that lacked a recombination event within the population [22, 23]. The genetic map included 1,522 bins that could be grouped into 13 sesame linkage groups (SLGs) with a total length of 1090.99 cM (Fig. 1, Additional file 1: Table S1). This is the first sesame genetic map in which the number of linkage groups equals the number of chromosome pairs in a sesame cultivar. The lengths of the 13 linkage groups ranged from 57.76 to 125.32 cM (mean value, 83.92 cM). Each linkage group had 84 (SLG12) to 168 (SLG3) bins resulting in a mean interval distance of 0.72 cM between adjacent bins (Additional file 1: Table S2). The bin distance was similar to that generated by RAD-Seq (0.69 cM) [5], but was shorter than that maps constructed using randomly selective amplification markers mainly (1.86 cM) [24] or the specific length amplified fragment sequencing (SLAF-seq) technology (1.20 cM) [4]. Furthermore, more than 99 % of the interval distances between adjacent bins were shorter than 6 cM (Additional file 2: Figure S1), indicating the high density of the new sesame genetic map.

### Update of sesame genome assembly

The previous *de novo* sesame assembly provided a total genome assembly of 274 Mb with a scaffold N50 of 2.1 Mb and 200 scaffolds greater than 150 kb in size. The assembly anchored 150 larger scaffolds into 16 pseudomolecules based on a genetic map that included 406 markers, which harbored 85.3 % of the total genome assembly and 91.7 % of the predicted genes (version 1.0) [2, 3]. Using the genetic map constructed in this study, we anchored another 172 scaffolds or contigs in a new sesame genome assembly (version 2.0) and linked together six smaller linkage groups in version 1.0 (LG12 and LG16, LG13 and LG14, and LG9 and LG15).

**Fig. 2:**
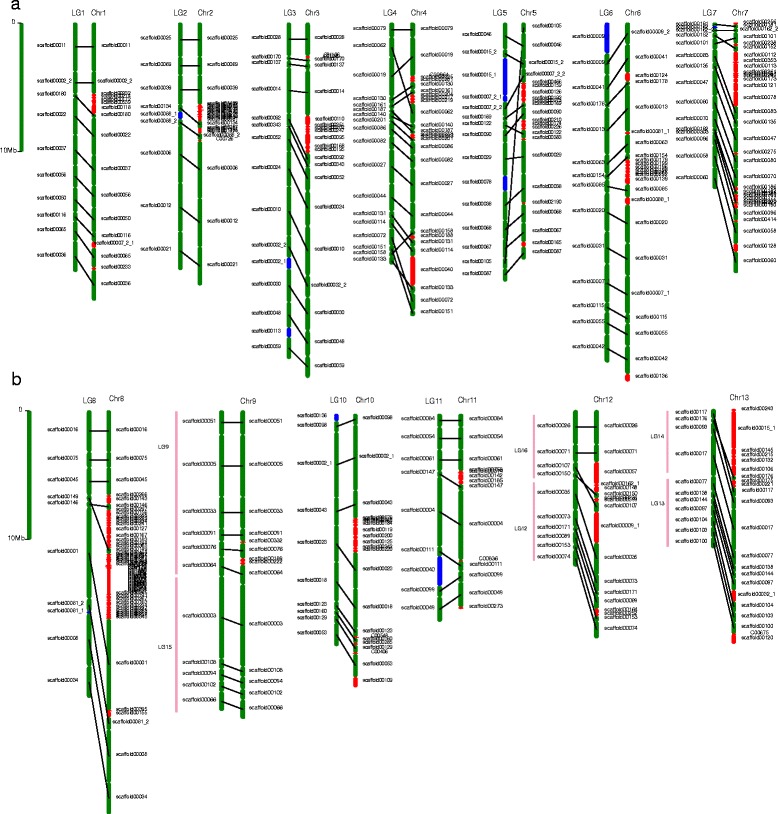
Anchored scaffolds in the current and previously assembled sesame genomes. Green scaffolds indicate anchored scaffolds that are consistent in the current (version 2.0) and previously (version 1.0) assembled genomes. Their relative positions are marked with lines. The newly anchored scaffolds in assembly version 2.0 are highlighted in red. The split scaffolds and relocated scaffolds are indicated in blue. The positions of the six smaller linkage groups in version 1.0 (LG9, LG12, LG13, LG14, LG15 and LG16) were indicated with pink lines

Despite the addition of 172 new scaffolds to the new version of the genome assembly, their orders were relatively consistent with the orders of the 150 scaffolds of version 1.0. In total, 20 changes occurred during the re-anchoring, including 10 scaffolds that were adjusted in the same pseudomolecules, 4 scaffolds that were relocated to other pseudomolecules, and 6 scaffolds that were split into two parts and relocated separately (Fig. 2; Additional file 1: Table S3 and S4). The inconsistency might be due to a single misassembly in which contig sequences were erroneously joined into these scaffolds through paired-end links during the initial assembly as reported for *Cucumis melo* [25, 26].

**Fig. 3:**
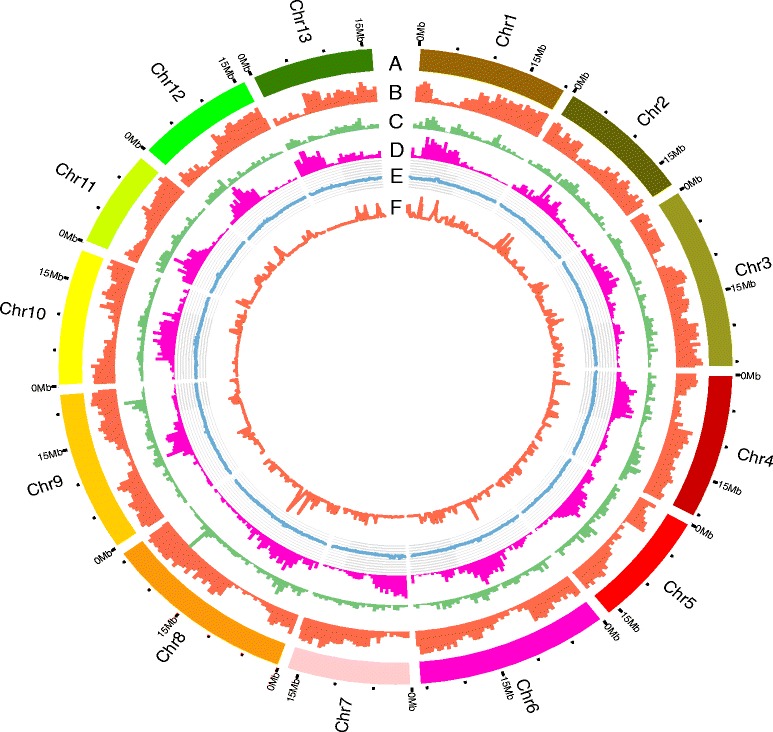
Distributions of basic elements of the sesame genome in the current assembly. **a** Pseudomolecules. **b** Gene density (mRNA); the frequency of sites within gene regions per 500 kb ranged from 0.04 to 0.61. **c** DNA transposon element (TE) density; the frequency of sites within the DNA TE regions per 500 kb ranged from 0 to 0.22. **d** Retrotransposon element density; the frequency of sites per 500 kb within the retrotransposon element regions ranged from 0 to 0.71. **e** GC content; the ratio of GC sites per 100 kb ranged from 0.32 to 0.40. **f** SNP density; the frequency of sites within SNP regions per 100 kb ranged from 1 × 10^−5^ to 1 × 10^−3^. Circos software (http://circos.ca) was used to construct the diagram

As a result, 327 scaffolds,(ncluding the split parts) were anchored to 13 pseudomolecules (Table 1), which were defined as pseudochromosomes and designated as Chr1 to Chr13 (Figs. 2 and 3). The lengths of the 327 anchored scaffolds ranged from 325 bp to 7.0 Mb (mean value, 784.7 kb; Additional file 2: Figure S2), 97.5 % of the scaffolds with lengths greater than 150 kb in version 1.0 were included in the new assembly version. The total length of the assembled pseudomolecules in the new version increased the assembly from 233.7 Mb to 258.4 Mb, representing 94.3 % of the genome assembly, and 97.2 % of the predicted gene models were anchored successfully (Fig. 3).

**Table 1:** Comparison of the anchored sesame genome versions 1.0 and 2.0

Version 2.0	V2.0_scaff_number	V2.0_size (Mb)	Version 1.0	V1.0_scaff_number	V1.0_size (Mb)
Chr1	17	20.26	LG1	10	18.58
Chr2	25	18.42	LG2	8	18.50
Chr3	22	25.85	LG3	14	24.93
Chr4	27	20.58	LG4	18	17.36
Chr5	23	16.58	LG5	13	18.90
Chr6	24	25.97	LG6	13	25.29
Chr7	34	16.76	LG7	14	11.73
Chr8	65	26.18	LG8	9	21.52
Chr9	14	22.85	LG9 & LG15	11	22.46
Chr10	22	19.49	LG10	10	17.25
Chr11	14	14.05	LG11	9	15.45
Chr12	18	16.28	LG12 & LG16	10	11.34
Chr13	22	16.47	LG13 & LG14	11	9.93
Total	327	259.73		150	233.22

There were 5 scaffolds (GenBank accession nos. KI866576.1, KI866583.1, KI866588.1, KI866606.1, and KI866610.1) with lengths greater than 150 kb that were still missing in the 13 newly assembled pseudomolecules. These sequences may represent conserved regions with few mutation sites or lower frequencies of exchange during chromosome recombination. Other high-density genetic maps based on new mapping populations or fluorescence *in situ* hybridization (FISH) may be required to anchor the remaining scaffold and to make the map more perfect. The mean sesame whole-genome recombination rate (cM/Mb) was 4.25; the rate ranged from 3.45 for Chr11 to 5.38 for Chr10. Comparison of the physical and genetic distances between markers revealed that most of the SLGs contained a low recombination region located approximately 5 Mb from the proximal end or center (Additional file 2: Figure S3).

### QTLs for traits related to sesame PH

**Fig. 4:**
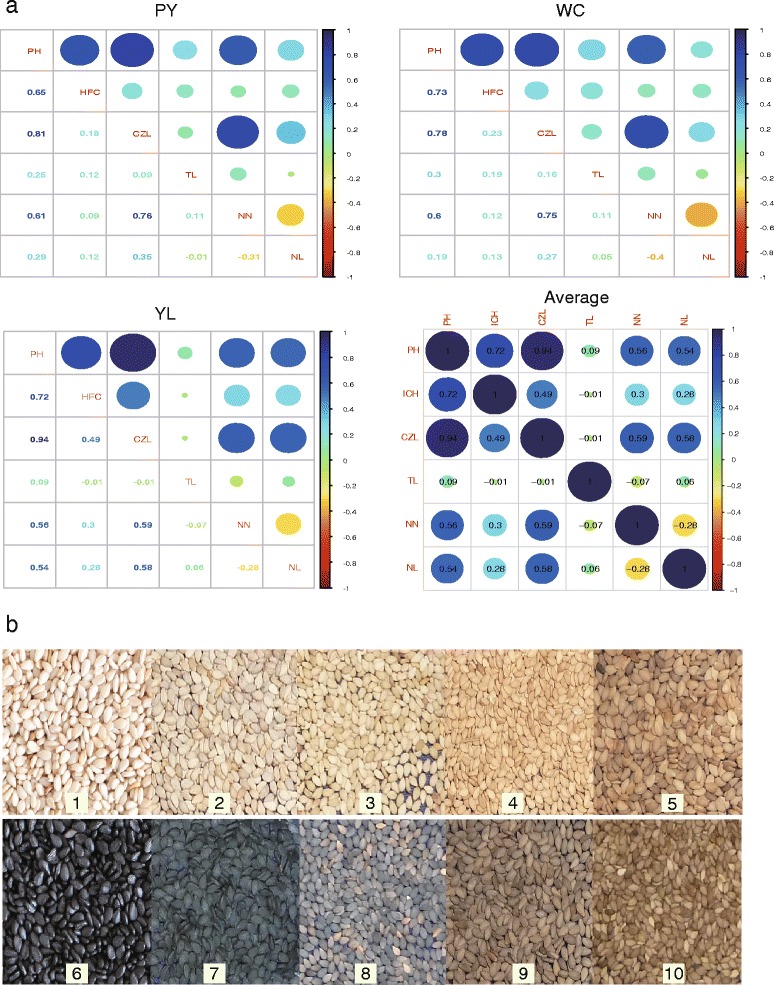
Characteristics of sesame plant height and seed coat color. **a** Correlations between sesame plant height and related traits across three trial sites. PH, plant height; CZL, capsule zone length; HFC, height of the first capsule-bearing node; IL, internode length; NN, node number; TL, tip length without the capsule; WC, Wuchang field trial location; PY, Pingyu field trial location; YL, Yangluo field trial location. The size and color of the ovals mirrored the correlation coefficient given in the lower triangle or ovals. **b** Variation in seed coat color in the RIL population. Subpanels 1 and 6 represent the seeds of the parental lines Zhongzhi No. 13 and ZZM2748, and the other panels represent the separation of the seed coat color in the population

The sesame plant height is generally determined by the height of the first capsule-bearing node (HFC), capsule zone length (CZL), and tip length without the capsule (TL) traits. The CZL is also related to the node number (NN) and internode length (IL) traits. The mature Zhongzhi No. 13 plants are normally greater than 1.8 m in height, whereas ZZM2748 is a semi-dwarf cultivar which grows to less than 0.9 m. Additionally, the two parents exhibited obvious differences in other traits related to PH (Additional file 2: Figure S4). Among the 430 progeny lines, the values for PH, HFC, CZL, TL, IL and NN were 0.65–1.96 (m), 0.15–1.16 (m), 0.22–1.28 (m), 0–15.8 (cm), 1.5–8.2 (cm) and 4.3–52.6, respectively (Additional file 2: Figures S5 and S6). PH, HFC, and IL fluctuated less than the other traits based on the coefficient of variation (*CV*) across the three field trials, and higher broad-sense heritabilities were also observed (Additional file 1: Table S5). Positive and negative transgressive segregation was observed in the RIL population suggesting that a germplasm could be created with the expected PH, CZL, TL, NN, and IL traits independent of environmental effects; however, the negative heterobeltiosis of HFC appeared to depend largely on the environment. PH was correlated with HFC, CZL, TL, NN, and IL (*P* < 0.01), and strong correlations were observed between PH and HFC and between CZL and NN (Fig. 4a). Interestingly, the correlation between NN and IL was negative across the three trial sites. This result supported the hypothesis that dwarf sesame cultivars could be bred without the loss of yield because reducing the internode length did not appear to affect the capsule node number.

Using the bin map, we identified QTLs underlying the sesame PH and the related traits HFC, CZL, TL, NN, and IL. Phenotypic datasets from the three experimental environments were coupled with the genotypic data using Windows QTL Cartographer 2.5 software [27]. The threshold used for the LOD scores to evaluate the statistical significance of the QTL effects was determined using 1,000 permutations. In total, 41 QTLs in nine SLGs were detected for the traits with contribution rates of 3–24 % (mean value, 8 %; Table 2, Fig. 1). Among them, 14, 15, and 12 QTLs were detected in three, two, and one environment(s), respectively. These loci were not distributed randomly because SLG3, SLG4, SLG8, and SLG9 together contained more than 78 % of the QTLs (Fig. 1).

**Table 2:** Significant QTLs* associated with sesame plant height and the seed coat color space

QTL	Linkage group	Position (cM)	Flanking bin_marker	Chr_space (kb)	LOD	Additive effect	R^2^	WC	PY	YL
qPH-3.1	SLG3	80.91	SLG3_bin98-SLG3_bin99	525.3	7.62	−5.17	0.06	✓	✓	✓
qPH-3.2	SLG3	92.41	SLG3_bin114-SLG3_bin115	735.1	20.75	−8.12	0.15	✓	✓	✓
qPH-3.3	SLG3	98.01	SLG3_bin126-SLG3_bin127	928.1	22.44	−8.91	0.18	✓	✓	✓
qPH-4.1	SLG4	34.11	SLG4_bin49-SLG4_bin50	103.0	10.58	5.86	0.08		✓	✓
qPH-4.2	SLG4	42.61	SLG4_bin56-SLG4_bin57	908.3	7.16	5.09	0.06	✓	✓	✓
qPH-8.1	SLG8	65.61	SLG8_bin107-SLG8_bin108	33.3	9.41	70.07	0.09	✓	✓	
qPH-8.2	SLG8	70.51	SLG8_bin111-SLG8_bin112	360.2	29.75	10.18	0.23	✓	✓	✓
qPH-9.1	SLG9	78.81	SLG9_bin102-SLG9_bin103	63.4	12.28	6.33	0.09		✓	✓
qPH-9.2	SLG9	84.71	SLG9_bin105-SLG9_bin106	1255.3	12.22	6.56	0.10	✓	✓	✓
qPH-12.1	SLG12	53.01	SLG12_bin66-SLG12_bin67	94.3	3.52	41.35	0.03	✓	✓	✓
qCZL-3.1	SLG3	99.00	SLG3_bin126-SLG3_bin127	928.1	10.43	−4.88	0.09			✓
qCZL-4.1	SLG4	34.00	SLG4_bin48-SLG4_bin49	127.4	7.95	4.09	0.07			✓
qCZL-4.2	SLG4	48.50	SLG4_bin59-SLG4_bin60	237.1	3.94	32.40	0.04	✓	✓	
qCZL-8.1	SLG8	71.50	SLG8_bin111-SLG8_bin112	360.2	27.69	7.82	0.24	✓	✓	✓
qCZL-9.1	SLG9	79.60	SLG9_bin103-SLG9_bin104	725.9	7.47	4.00	0.06		✓	✓
qCZL-10.1	SLG10	57.60	SLG10_bin104-SLG10_bin105	176.6	2.78	2.39	0.02			✓
qCZL-12.1	SLG12	52.20	SLG12_bin65-SLG12_bin66	101.8	3.90	2.83	0.03	✓	✓	✓
qCZL-13.1	SLG13	24.40	SLG13_bin32-SLG13_bin33	110.9	6.65	42.23	0.07	✓	✓	
qHFC-3.1	SLG3	92.21	SLG3_bin112-SLG3_bin113	324.9	9.99	−34.32	0.09		✓	
qHFC-3.2	SLG3	97.01	SLG3_bin126-SLG3_bin127	928.1	23.26	−3.65	0.22	✓	✓	✓
qHFC-4.1	SLG4	32.41	SLG4_bin45-SLG4_bin46	359.2	6.13	1.75	0.05	✓	✓	✓
qHFC-8.1	SLG8	65.71	SLG8_bin107-SLG8_bin108	33.3	5.59	30.10	0.05	✓	✓	
qHFC-8.2	SLG8	69.51	SLG8_bin111-SLG8_bin112	360.2	10.91	2.37	0.09	✓	✓	✓
qHFC-9.1	SLG9	78.81	SLG9_bin102-SLG9_bin103	63.4	12.40	2.46	0.10		✓	✓
qHFC-9.2	SLG9	84.71	SLG9_bin105-SLG9_bin106	1255.3	12.96	2.58	0.11			✓
qHFC-9.3	SLG9	91.01	SLG9_bin112-SLG9_bin113	78.7	12.40	2.48	0.10		✓	✓
qIL-3.1	SLG3	41.10	SLG3_bin53-SLG3_bin54	153.7	7.57	0.15	0.07		✓	✓
qIL-8.1	SLG8	71.50	SLG8_bin111-SLG8_bin112	360.2	10.52	0.17	0.09	✓	✓	✓
qIL-9.1	SLG9	83.70	SLG9_bin105-SLG9_bin106	1255.3	9.76	0.17	0.10	✓	✓	✓
qIL-12.1	SLG12	53.50	SLG12_bin69-SLG12_bin70	63.2	3.86	1.61	0.04	✓		✓
qNN-3.1	SLG3	91.80	SLG3_bin111-SLG3_bin112	193.0	4.62	−0.91	0.05	✓		
qNN-3.2	SLG3	100.10	SLG3_bin126-SLG3_bin127	928.1	10.15	−1.31	0.10			✓
qNN-4.1	SLG4	54.00	SLG4_bin72-SLG4_bin73	466.0	3.96	0.94	0.04	✓	✓	
qNN-8.1	SLG8	66.50	SLG8_bin110-SLG8_bin111	1154.1	7.99	1.13	0.07		✓	✓
qNN-13.1	SLG13	24.40	SLG13_bin32-SLG13_bin33	110.9	8.75	1.42	0.09	✓	✓	
qTL-2.1	SLG2	66.10	SLG2_bin93-SLG2_bin94	686.3	3.04	4.68	0.03		✓	
qTL-3.1	SLG3	94.40	SLG3_bin124-SLG3_bin125	75.7	7.90	−7.68	0.08		✓	
qTL-3.2	SLG3	103.60	SLG3_bin131-SLG3_bin132	83.2	7.38	−6.58	0.08	✓		
qTL-4.1	SLG4	74.10	SLG4_bin93-SLG4_bin94	169.7	3.04	0.36	0.03			✓
qTL-5.1	SLG5	35.60	SLG5_bin58-SLG5_bin59	71.1	3.19	−4.24	0.03	✓		
qTL-13.1	SLG13	8.80	SLG13_bin22-SLG13_bin23	1362.3	2.67	−0.35	0.03		✓	✓
qSCa-4.1	SLG4	50.90	SLG4_bin63-SLG4_bin64	199.9	15.41	0.84	0.13	✓	✓	
qSCa-8.1	SLG8	73.40	SLG8_bin114-SLG8_bin115	2518.0	26.73	1.18	0.25	✓	✓	
qSCa-8.2	SLG8	62.60	SLG8_bin105-SLG8_bin106	575.8	9.02	0.69	0.09		✓	
qSCb-4.1	SLG4	50.90	SLG4_bin63-SLG4_bin64	199.9	54.75	4.81	0.39	✓	✓	
qSCb-8.1	SLG8	72.40	SLG8_bin114-SLG8_bin115	2518.0	27.08	3.11	0.21	✓	✓	
qSCb-11.1	SLG11	0.00	SLG11_bin1-SLG11_bin2	367.8	5.56	1.38	0.03	✓	✓	
qSCl-4.1	SLG4	50.90	SLG4_bin63-SLG4_bin64	199.9	30.34	5.62	0.21	✓	✓	
qSCl-8.1	SLG8	73.40	SLG8_bin114-SLG8_bin115	2518.0	44.06	7.14	0.46	✓	✓	
qSCl-11.1	SLG11	0.00	SLG11_bin1-SLG11_bin2	367.8	20.76	4.64	0.14	✓	✓	

* Traits related to plant height including capsule zone length (CZL), height of the first capsule-bearing node (HFC), internode length (IL), node number (NN), and tip length without the capsule (TL) are also listed. “Flanking bin_marker” indicates the bin_markers flanking the related QTL; “Chr_space” indicates the distance between the flanking bin_markers on the sesame chromosome; “Additive effect” indicates the estimated value for the genotype transmitted stably to offspring; “R^2^” indicates the contribution rate of the locus to the phenotype; Check marks in the last three columns indicate that the QTL was detected at a specific trial site; WC, Wuchang field trial location; PY, Pingyu field trial location; YL, Yangluo field trial location

Ten QTLs accounting for 3.03–23.32 % of the PH variation were detected. Four of these QTLs (*qPH-3.2*, *qPH-3.3*, *qPH-8.2,* and *qPH-9.2*) individually explained more than 10 % of the phenotypic variation (Table 2). We also detected eight HFC, eight CZL, and six TL QTLs. Of them, three of the HFC QTLs (*qHFC-3.2*, *qHFC-9.1*, and *qHFC-9.3*) and one CZL QTL (*qCZL-8.1*) were found to contribute more than 10 % of the phenotype variation at a minimum of two sites. TL might be easily affected by the environment because all of the TL QTLs were detected in only one or two of the three trial sites. Although five QTLs were detected for NN, none of them had a phenotype explanation rate of more than 10 %. Nevertheless, the *qNN*-13.1 locus accounted for approximately 9 % of NN and had a pleiotropic effect on CZL (*qCZL-13.1*) with a contribution rate of 6.89 %.

We also identified loci with pleiotropic effects in other QTLs based on their close or identical locations, particularly in QTLs with higher contribution rates. The *qPH-8.2* and *qPH-3.3* QTLs were the two major loci that accounted for 23.03 and 18.07 % of the sesame PH variation, respectively. The *qPH-8.2* locus was derived from the tall parent with a positive effect of 10.18 % and was located between bin markers SLG8_bin111 and SLG8_bin112 in a 350-kb region. This region also contained three other QTLs (*qHFC-8.2*, *qCZL-8.1*, and q*IL-8.1*) that accounted for 8.91, 23.84, and 9.45 % of the phenotypic variation in HFC, CZL, and IL, respectively. The *qPH-3.3* locus was located in a 928-kb region between bin126 and bin127 on SLG3. This region also contained three other QTLs (*qHFC-3.2*, *qCZL-3.1*, and *qNN-3.2*) that accounted for 22.00, 9.33, and 10.26 % of the phenotypic variation in HFC, CZL, and NN, respectively. The negative additive effects of these QTLs indicated that the ZZM2748 semi-dwarf parent contributes to a strong decrease in the sesame PH and related traits including HFC, CZL, and NN.

Plant height usually decides the plant architecture and contributes to the crop yield. Studies based on mutants with dwarf phenotypes had showed that plant hormones such as gibberellin (GA), auxin, cytokinin (CK), and brassinosteroid (BR) played important roles in determining stem elongation [28, 29]. In sesame, few studies have reported the locus or gene that encodes plant height; the exception is Wu et al.[5], who he detected two QTL with contribution rate no more than 6. However, the relationships of these QTLs were not clear for the inconsistent genetic maps and no sesame reference genomes available for the previous study. We predicted 53 and 102 candidate genes for the flanking bin-marker regions of *qPH-8.2* and *qPH-3.3*, respectively, on the new assembled pseudomolecule chromosome according to the true confidence intervals (Additional file 1: Table S6). Annotation of these genes showed some of them may function in regulating of plant height. For example, SIN_1015931 in the site of *qPH-3.3* site was predicted to encode an auxin-induced protein and SIN_1015910 encoded a brassinosteroid-insensitive protein. Further study in the future may be expected to verify their functions.

### QTL for sesame seed coat color

The two parents Zhongzhi No. 13 and ZZM2748 were also distinguishable by seed coat color; the seeds of the former were white and the seeds of the latter were black (Fig. 4b). Genetic analysis showed that black was dominant to white in sesame seed coat color. The black seed coat color was characterized by delayed inheritance or predetermination because the F1 seeds (F2 organs in theory) produced from a cross between white and black seed sesame plants were always black. The RILs exhibited wide variations in seed coat color. The L*, a*, and b* values for the Zhongzhi No. 13 seed coat color were 64.35, 3.84, and 20.21, respectively, whereas those for ZZM2748 were 25.34, 0.73, and 2.87, respectively. The L* values for the 430 RILs ranged from 18.16 to 65.69 and the a* and b* values ranged from −0.12 to 12.62 and from 0.74 to 55.77, respectively (Additional file 2: Figure S7). Because “L*” represents brightness ranging from black to white, “a*” represents red and “b*” represents yellow for positive values [30], the measured values and distributions suggest that black, white, and yellow are predominant in the sesame seed coat color space, which is consistent with the observation that the seed coat color segregates in the RILs.

Across the Pingyu (PY) and Yangluo (YL) field trial locations, three QTLs (*qSCl-8.1*, *qSCl-4.1*, and *qSCl-11.1*) were detected repeatedly for the L* color space and made a cumulative contribution of 80 % to the phenotype. Among them, *qSCl-8.1* explained approximately 46.0 % of the L* variation and was located at 73.40 cM on the SLG8 linkage group. This locus was flanked by the adjacent SLG8_bin114 (72.4 cM) and SLG8_bin115 (80.4 cM) bin markers that covered a 2.5-Mb region on pseudochromosome Chr8. The *qSCl-4.1* locus was located in a 199.9-kb region on pseudochromosome Chr4 between the bin markers SLG4_bin63 (50.4 cM) and SLG4_bin64 (50.9 cM) and contributed 20.6 % of the L* variation. The *qSCl-11.1* locus was located at the top of pseudochromosome Chr11 in a 367.8-kb region between bin markers SLG11_bin1 (0 cM) and SLG11_bin2 (0.15 cM) and explained 13.7 % of the L* variation. There two QTLs (*qSCa-8.1* and *qSCa-4.1*) contributed 25.3 and 13.2 % of the color space a* value detected across the PY and YL field trial locations, respectively. Locus *qSCa-8.2* was detected only at PY with a phenotype explanation rate of 9.2 %. Three QTLs (*qSCb-4.1*, *qSCb-8.1*, and *qSCb-11.1*) were detected for the color space b* across both locations with a total contribution rate of 64 %. The high contribution rate of the seed coat color locus to phenotype was also reported by Zhang et al., and he detected four QTL with contribution rates ranging from 9.6 to 39.9 % [24]. However, because the loci in the study of Zhang et al. were mainly located with AFLP markers in an independent genetic map, it is difficult to determine the relationship of the present loci to them.

Of the QTLs described above, *qSCl-8.1*, *qSCa-8.1*, and *qSCb-8.1* were located in the same region on pseudochromosome Chr8, the *qSCl-4.1*, *qSCb-4.1* and *qSCa-4.1* QTLs were located in the same region on pseudochromosome Chr4, and *qSCl-11.1* and *qSCb-11.1* were located in the same region on pseudochromosome Chr11; thus, the three sites were pleiotropic for the sesame seed coat color. In other words, only 4 loci were detected here. In contrast, the intricate manifestation of seed coat color has been acknowledged to be due to the involvement of various pigments including flavonols, proanthocyanidin (carotenoid content condensed tannin), and possibly other phenolic relatives such as lignin and melanin [31]. Polyphenol oxidase (*PPO*) was reported to participate in the oxidation step in proanthocyanidin, lignin, and melanin biosynthesis, resulting in a dark seed coat color [32, 33]. Some genes encoding the enzymes participating in flavonoid biosynthesis have been cloned from Arabidopsis [34], grape [35], and soybean [36]. We checked the two sites of *qSCl-8.1* and *qSCl-4.1* with higher explanation rates (Additional file 1: Table S7 and S8) and found that the gene that encoded *PPO* (SIN_1016759), which was reported to account for the black color of the sesame seed coat, was also located at *qSCl-4.1* [37]. For site *qSCl-8.1*, a gene (SIN_1022830) belonging to the cytochrome family that contained a ferredoxin reductase-type FAD-binding domain and flavoprotein transmembrane component was identified; this gene may contribute to the seed coat color [38]. These results also highlight the availability of the genome assembly.

## Conclusions

No previously reported sesame genetic map has contained the same number of linkage groups as the number of chromosomes. The high number of linkage groups (even in the sesame genome assembly version 1.0) has caused confusion in the numbering of linkage groups and the assembled pseudomolecules in various studies. The present study brought the total number of assembled pseudomolecules equals the chromosome number of *Sesamum indicum*, and that 94.3 % of the *de novo* assembly and 97.2 % of the predicted gene models were anchored successfully. Thus, the number of sesame pseudochromosomes is fixed, which is important and helpful for comparative genomics and genetics studies such as sesame genome resequencing, marker development, genetic map integration, gene mapping, allele analysis, and evolutionary analyses.

We provide a high-density sesame bin map containing 1,522 bins spanning 1090.99 cM. The map covers the entire assembled genome and is useful for mapping QTLs or genes. Aside from the investigation of traits important for breeding sesame cultivars, such as indehiscent capsule, definite growth habit, PH, and seed coat color [24, 39, 40], few agronomic traits have been studied in the sesame. The present study not only mapped 41 QTLs for sesame PH and related traits but also detected nine QTLs for sesame seed coat color. In particular, two major QTLs (*qPH-8.2* and *qPH-3.3*) associated with PH were located in a 350-kb region on Chr8 and in a 928-kb region on Chr3, respectively. We identified 53 and 102 candidate genes, respectively, in the two regions for future study. Notably, the *qPH-3.3* locus is predicted to be responsible for the semi-dwarf sesame plant phenotype. This is the first report of a sesame semi-dwarf locus that can be used in an architectural study of sesame. The major *qSCl-8.1* pleiotropic seed coat color locus for the L*, a*, and b* color space values was located on Chr8 in a 2.5-Mb region. Another major locus *qSCb-4.1* located on Chr4 in a 199.9-kb region contributed 39 % to the b* values and contained 32 candidate genes. Collectively, the combination of the high-density genetic map and the sesame genome assembly update will serve as an essential platform for future comparative genomics and genetic studies.

## Methods

### Plant material and field trials

The ZZM2748 semi-dwarf sesame strain with a black seed coat was discovered and identified from among the 6,000 sesame germplasms preserved at the National Medium-Term Sesame Genebank of China (Wuhan, China). The mapping population consisted of 430 recombinant inbred lines (RILs, F8) and was derived from a cross between the Zhongzhi No. 13 (female parent) and the semi-dwarf ZZM2748 (male parent) cultivars. Zhongzhi No. 13 was *de novo* sequenced in 2014 [2] and was characterized by a high plant height (PH) and white seed coat color.

Three field trials were conducted in 2013 (F8) and 2014 (F8:9) during the normal sesame planting season (June–September) in three environments (Wuchang (2013), Pingyu (2014), and Yangluo (2014)) in China to evaluate trait variations in the RIL population. The 430 lines were grown in a randomized complete block design with three replicate plots in each trial. Each trial plot comprised three 2-m rows spaced 40 cm apart with a plant spacing of 10 cm. Sesame PH and related traits including height of the first capsule-bearing node (HFC), capsule zone length (CZL), tip length without the capsule (TL), node number (NN), and internode length (IL) were measured in each plot at a stage near maturity.

The seeds harvested from the Pingyu (2014) and Yangluo (2014) trial location environments were used to evaluate the sesame seed coat color. The seeds from three replicate plots of each line at each site were mixed together and evaluated using a Chroma Meter CR-400 (Konica Minolta, Japan). The Chroma Meter measures color by decomposing it into the L*, a*, and b* color space values, where “L*” represents brightness (0 for black, 100 for white), “a*” represents the color red when positive and the color green when negative, and “b*” represents the color yellow when positive and the color blue when negative [30].

### DNA extraction and restriction-site associated DNA (RAD) sequencing

Healthy young leaves were collected from the two parental lines and the 430 RILs (F8) in 2013 and used for DNA extraction. The cetyltrimethylammonium bromide (CTAB) method was used with modifications to prepare total genomic DNA [4, 41]. DNA concentrations and quality were estimated using an ND-1000 spectrophotometer (NanoDrop, Wilmington, DE, USA) and 0.8 % agarose gel electrophoresis with a lambda DNA standard [4].

RAD sequencing was performed according to the method of Baird et al. with some modifications [20]. Briefly, the extracted DNA was digested with the *Taq* I restriction enzyme (Takara, Dalian, China), and adapters containing a multiplex identifier (MID) were added to the samples. The ligation products were combined in appropriate multiplex pools (either two parental samples or seven RIL samples per library pool). Each of the library samples was size-selected by gel electrophoresis for DNA fragments that were 350–600 bp in length. Finally, the libraries were enriched by PCR amplification, quantified on an Agilent 2100 Bioanalyzer (Agilent Technologies, Santa Clara, CA, USA), and sequenced on an Illumina HiSeq2000 instrument (San Diego, CA, USA) using paired-end reads (115 bp).

### Sequence analysis and SNP discovery

Raw reads from multiple Illumina sequence channels were separated using the appropriate MID and assigned to each sample. The reads without unique barcodes and those with low quality in which 10 % of the nucleotides had a quality value less than Q30 (equals to 0.1 % sequencing error) were discarded. The clean pair-end reads were further trimmed to the RAD tags with a uniform length of 94 nucleotides.

All high-quality reads were aligned against the Zhongzi No. 13 scaffold sequences using BWA software [21]. The “mpileup” function in SAMtools software [42] was used to detect the SNPs between the Zhongzhi No. 13 and ZZM2748 parental lines using reads with mapping quality values greater than or equal to 20. The detected SNPs were assessed in the RILs and used for genotyping. The RIL genotypes were assigned to the Zhongzhi No. 13 or ZZM2748 homozygous genotypes or scored as heterozygous if the genotype was the same as both parents.

### SNP marker development and linkage map construction

Prior to the construction of the linkage map, low quality SNP markers that were not homozygous in both parents or with a proportion of missing data greater than 70 % in the RIL population were filtered out. For each scaffold, 1 to 30 high-quality SNPs were selected from the head, middle and end. The segregation ratios of the SNP markers were evaluated using the chi-square test, and significantly distorted (*P* < 0.01) markers were removed. The remaining SNPs were grouped and ordered according to pairwise recombination frequencies at a logarithm of minimum odds (LOD) of 4.0 using JoinMap 4 software [43]. The Kosambi mapping function was chosen to translate the recombination frequencies into map distances in centiMorgans (cM). The goodness-of-fit of the calculated regression map for each tested position was checked against the default parameter. When the SNPs from the same scaffold were dispersed by other SNP on the map, all of the SNPs on the interactive scaffolds were regrouped and reordered and the related scaffolds were split at the recombinant breakpoints. The scaffolds were anchored to the pseudomolecules by relying on the order of the SNPs in the primary genetic map.

The bin map was constructed after the scaffolds were anchored according the method of Huang et al.[23]. Briefly, the consecutive genotypic SNPs on each pseudomolecule were scanned with a sliding window size of 15 SNPs and a step size of 1. For each window, the SNP from either parent were calculated. The windows containing 13 or more SNPs from either parent were considered to be homozygous for an individual, whereas those with less were classified as heterozygous. Adjacent windows with the same genotype were combined into blocks based on the recombinant breakpoints, and the blocks were considered to be bins [22, 44]. The bins on each pseudomolecule were taken as genetic markers and analyzed for linkage groups using JoinMap 4 with the setup described above. Finally, a sesame bin map was constructed.

### QTL analysis

The frequency distributions of the mean phenotypic data for all 430 lines in each trial were analyzed using the R package software (https://www.r-project.org/). The QTLs underlying the six traits were detected using the composite interval mapping method implemented in Windows QTL Cartographer 2.5 software (Microsoft, Inc. Redmond, WA, USA) [27]. A 1,000 permutation parameter was set to determine the statistical significance of the QTL effects and a minimum LOD score of 2.5 was used to judge the presence of a QTL. QTLs were named according to the trait and linkage group locations. QTL names were designated based on the rules of the wheat gene nomenclature (http://wheat.pw.usda.gov/ggpages/wgc/98/Intro.htm).

### Availability of supporting data

The RAW data of RAD-seq had been submitted to NCBI under the BioProject PRJNA301193 with SRA accession number SRA308937. The data sets supporting the results of this article including the updated genome assembly and annotation are available in http://ocri-genomics.org/Sinbase_v2.0 (genome assembly) and http://ocri-genomics.org/Sin_SNP_430RIL.tar.gz (SNP information). The candidate gene sequences near the mapped QTL can be accessed from sesame genome database (http://ocri-genomics.org/Sinbase).

## Additional files

Additional file 1:
**Consists of Tables S1–S8.** **Table S1.** Information and relationships of the sesame high-density genetic map to genome assembly; **Table S2.** Bin marker information for the high-density genetic map; **Table S3.** List of the scaffolds anchored onto chromosomes; **Table S4.** The 6 split scaffolds and the 4 relocated scaffolds; **Table S5.** Variation in plant height and related traits in the population across field trials. **Table S6.** List of genes in QTLs *qPH-8.2* and *qPH-3.3*; **Table S7.** List of genes in the *qSCl-8.1* QTL; **Table S8.** List of genes in the *qSCb-4.1* QTL. (XLSX 152 kb) Additional file 2:
**Consists of Figures S1–S7.** **Figure S1.** Distributions of the interval distances between adjacent markers on the genetic map; **Figure S2.** Length distributions of the anchored scaffolds; **Figure S3.** Genetic distance vs. physical distance. The genetic position of the markers was plotted against the corresponding physical position; **Figure S4.** Variations in sesame plant height and the height of the first capsule-bearing node in the two parents and the RILs; **Figure S5.** Boxplots of sesame plant height and related traits of the population across three trial sites; **Figure S6.** Histograms of the segregation of sesame plant height and related traits across three trial sites; **Figure S7.** Distributions of the L*, a*, and b* color space values across two trial sites. (DOCX 1406 kb)

## Abbreviations

CZL: capsule zone length
FISH: fluorescent in situ hybridization
HFC: height of the first capsule-bearing node
IL: internode length
LOD: logarithm of odds
MID: multiplex identifier
NN: node number
PH: plant height
PY: Pingyu field trial location
QTL: quantity trait loci
RAD: restriction-site associated DNA
SLAF: specific length amplified fragment
SLG: sesame linkage groups
TL: tip length without capsule
WC: Wuchang field trial location
YL: Yangluo field trial location
