# Supplementary material for: Updated sesame genome assembly and fine mapping of plant height and seed coat color QTLs using a new high-density genetic map
Source: BMC Genomics. 2016 Jan 5;17:31. doi: 10.1186/s12864-015-2316-4 (PMC4702397; doi:10.1186/s12864-015-2316-4)
Supplement: Additional file 2: — Consists of Figures S1–S7. Figure S1. Distributions of the interval distances between adjacent markers on the genetic map; Figure S2. Length distributions of the anchored scaffolds; Figure S3. Genetic distance vs. physical distance. The genetic position of the markers was plotted against the corresponding physical position; Figure S4. Variations in sesame plant height and the height of the first capsule-bearing node in the two parents and the RILs; Figure S5. Boxplots of sesame plant height and related traits of the population across three trial sites; Figure S6. Histograms of the segregation of sesame plant height and related traits across three trial sites; Figure S7. Distributions of the L*, a*, and b* color space values across two trial sites. (DOCX 1406 kb) [file 12864_2015_2316_MOESM2_ESM.docx]

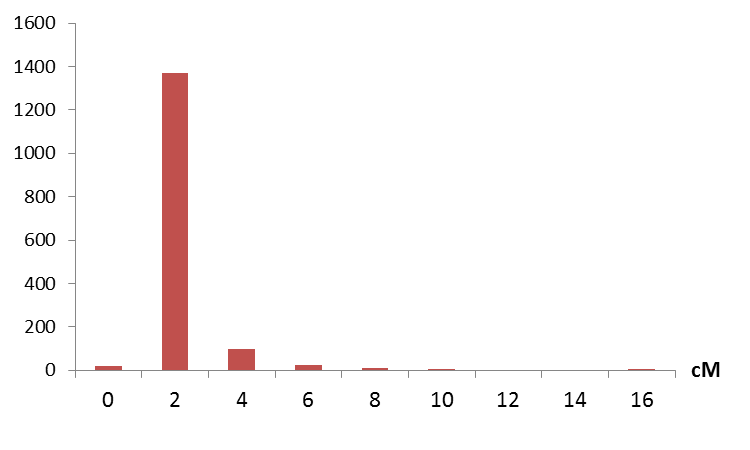


Figure S1. Distributions of the interval distances between adjacent markers on the genetic map.

Figure S2. Length distributions of the anchored scaffolds.

**Physical distance** (Mb)


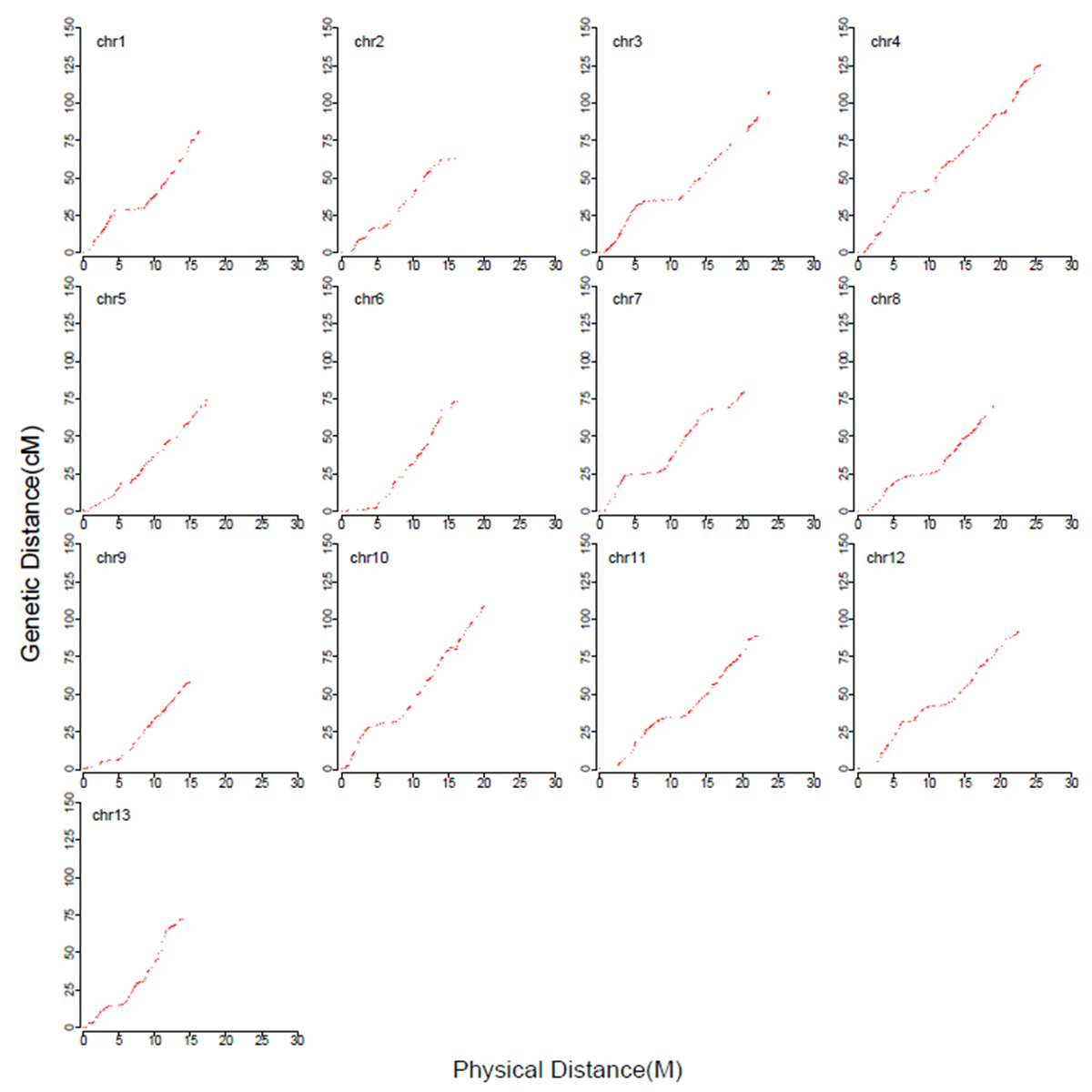


Figure S3. Genetic distance vs. physical distance. The genetic position of the genetic markers was plotted against the corresponding physical position.

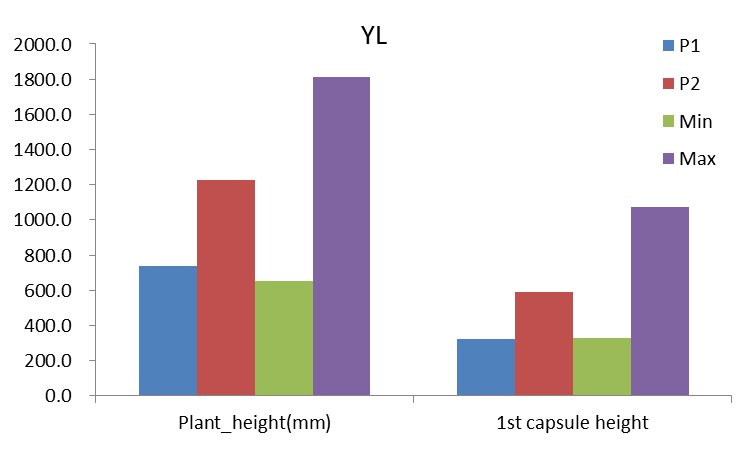


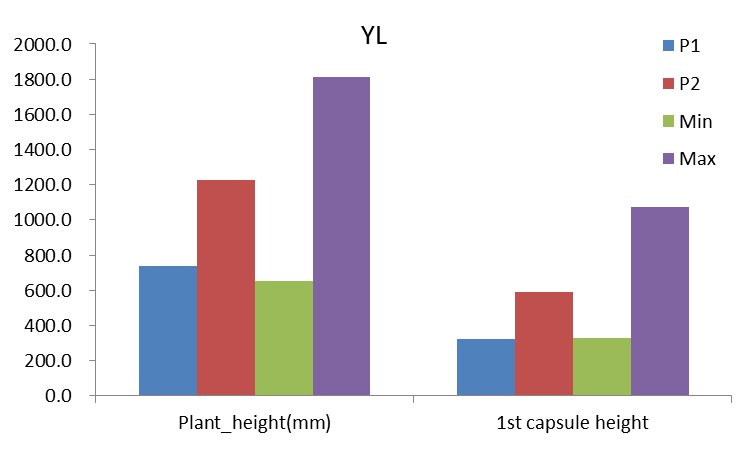


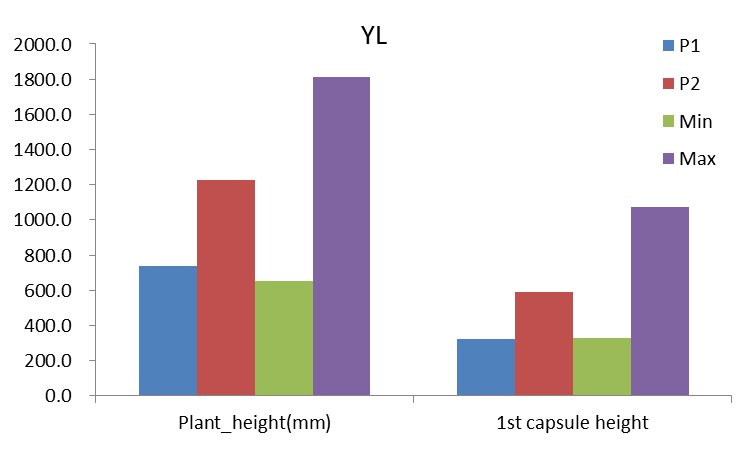


Figure S4. Variations in sesame plant height and the height of the first capsule-bearing node in the two parents and RILs.

Plant height

Height of 1st capsule-bearing node

Tip length without capsule

Internode length

Capsule zone length

Node number

Figure S5. Boxplots of sesame plant height and related traits of the population across three trial sites. WC:Wuchang; PY: Pingyu; YL: Yangluo.

Figure S6.Histograms of the segregation of sesame plant height and related traits across three field trials. WC:Wushang; PY: Pingyu; YL: Yangluo.

Figure S7. Distributions of the L*, a*, and b* color space values across two trial sites. PY: Pingyu; YL: Yangluo.
